# Association between C-reactive protein-albumin-lymphocyte index and stroke: an NHANES analysis (1999–2010)

**DOI:** 10.3389/fneur.2025.1548666

**Published:** 2025-04-02

**Authors:** Yizhou Chen, Meifang Liu, Yi Zhang, Xiaolin Yang, Mengqi Yue, Xu Chen, Haiqiang Wang, Zirong Wang, Haocheng Yu, Jing Shi

**Affiliations:** ^1^Yunnan University of Traditional Chinese Medicine, Kunming, Yunnan, China; ^2^First Affiliated Hospital of Yunnan University of Traditional Chinese Medicine, Kunming, Yunnan, China; ^3^Yunnan Provincial Hospital of Traditional Chinese Medicine, Kunming, Yunnan, China; ^4^Qingdao Central Hospital, University of Health and Rehabilitation Sciences, Qingdao, Shangdong, China

**Keywords:** stroke, C-reactive protein-albumin-lymphocyte index, NHANES, inflammation, CALLY index

## Abstract

**Objective:**

This cross-sectional study is based on the NHANES (1999–2010) database and aims to explore the potential relationship between the CALLY index and stroke in the U.S. population.

**Methods:**

This cross-sectional study utilized data from NHANES (1999–2010), including 17,511 American participants after data cleaning. Laboratory markers related to the CALLY index were obtained through standardized biological sample collection and analysis procedures performed by trained professionals. Stroke status was determined based on self-reported questionnaires. Various statistical methods were employed to examine the association between the CALLY index and stroke, as well as its predictive efficacy for stroke risk, including multivariable logistic regression, subgroup analysis, RCS analysis, and ROC analysis.

**Results:**

Among the 17,511 participants analyzed, our findings revealed a nonlinear L-shaped negative association between the CALLY index and stroke risk. In Model 3, a higher CALLY index was significantly associated with a lower stroke risk (OR: 0.99, 95% CI: 0.98–0.99, *p* = 0.045). Additionally, participants in the highest quartile (Q4) of the CALLY index had a 25% lower likelihood of stroke compared to those in the lowest quartile (Q1) (OR: 0.75, 95% CI: 0.58–0.97, *p* = 0.030). Furthermore, ROC analysis demonstrated that the CALLY index had superior predictive performance for stroke risk compared to the SIRI and SII indices.

**Conclusion:**

A reduced CALLY index may be linked to a higher risk of stroke. Furthermore, the CALLY index demonstrates superior predictive performance compared to the SIRI and SII indices. The association between the CALLY index and stroke risk provides valuable insights for future stroke prevention and management strategies.

## 1 Introduction

Stroke, also referred to as a cerebrovascular accident (CVA), is a leading cause of mortality and disability worldwide. Stroke is classified into ischemic stroke (IS) and hemorrhagic stroke (HS), with ischemic stroke comprising more than 70% of cases. The incidence of ischemic stroke has been rising annually, with an increasingly younger age of onset (1). Hemorrhagic stroke is a severe condition resulting from the rupture of cerebral blood vessels, leading to localized mechanical and compressive damage (2). If left untreated, ischemic stroke and cerebral ischemia can lead to severe neurological dysfunction or even death. Numerous studies have demonstrated that both types of stroke involve blood–brain barrier disruption, inflammatory cytokine release, and immune cell infiltration, with immune cells and inflammatory responses playing critical roles in stroke pathogenesis (3–5).

Studies have demonstrated that the Systemic Inflammation Response Index (SIRI) and the Systemic Immune-Inflammation Index (SII) are strongly associated with stroke onset and prognosis (6–8). The C-reactive protein–albumin–lymphocyte (CALLY) index is a newly developed inflammation-based biomarker. It consists of the inflammatory index (C-reactive protein), nutritional index (serum albumin), and immune index (lymphocytes), which reflect inflammation levels, nutritional status, and immune function, respectively (9). Previously, it has been mainly utilized for postoperative prognosis assessment in patients with solid tumors, such as liver cancer (10), gastric cancer (11), breast cancer (12), and oral cancer (13). As a composite index, it offers a more comprehensive assessment of the patient’s immune status and systemic inflammatory balance, exhibiting significant advantages in tumor prognosis prediction. In stroke pathophysiology, inflammation, immune responses, and nutritional status play pivotal roles. The CALLY index, as a composite biomarker, reflects systemic inflammation, immune status, and nutritional condition. Therefore, it serves as a promising biomarker for predicting stroke risk and guiding early interventions and preventive strategies. However, studies investigating the CALLY index in neurological diseases remain limited. This study thus aims to explore the association between the CALLY index and stroke and further evaluate its predictive value for stroke occurrence.

## 2 Methods

### 2.1 Study population

**Figure 1:**
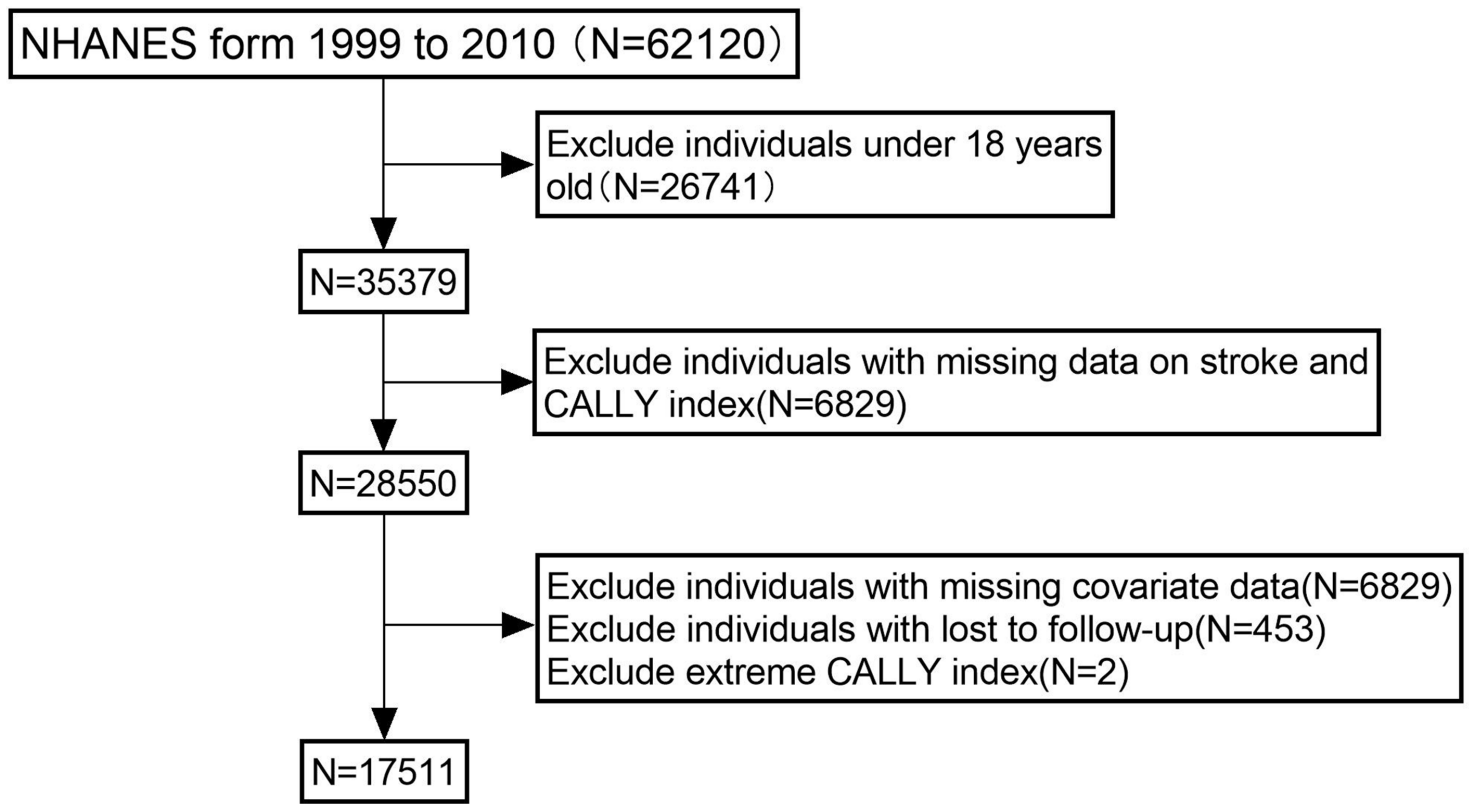
Flowchart of the sample selection from NHANES 1999–2010.

The data for this study were obtained exclusively from the NHANES database,^1^ a nationally representative cross-sectional survey conducted by the Centers for Disease Control and Prevention (CDC) in collaboration with the National Center for Health Statistics (NCHS) (14). The NHANES database collects demographic, health status, laboratory, and nutritional data through a complex multistage probability sampling design, incorporating household interviews, mobile examination centers, and biospecimen collection. The study protocol was approved by the NCHS Research Ethics Review Board, and informed consent was obtained from all participants (15). This study included NHANES data from 1999 to 2010 for cross-sectional analysis. The objective was to investigate the association between the CALLY index and stroke. Initially, individuals aged 18 years or older from six consecutive NHANES cycles (1999–2010) were included, yielding a total sample size of 35,379. Participants were excluded if they had missing data on stroke or CALLY index-related variables (*N* = 6,829), lacked covariate data (*N* = 10,584), were lost to follow-up (*N* = 453), or were identified as extreme outliers (*N* = 2). The final analytical sample comprised 17,511 participants. Detailed information is shown in Figure 1.

### 2.2 Definitions of stroke, CALLY index, SIRI index, and SII index

In this study, stroke diagnosis was primarily determined based on self-reported data from personal interviews. Participants completed a questionnaire in which they reported whether a physician or other healthcare professional had diagnosed them with a stroke (16). Laboratory parameters related to the CALLY, SIRI, and SII indices were measured by trained professionals using standardized biospecimen collection and analysis protocols. The calculation formulas for the CALLY, SIRI, and SII indices are as follows (17–19): CALLY = Serum albumin level (g/dL) × Lymphocyte count (1,000 cells/mL)/C-reactive protein (mg/dL) /10; SIRI = Neutrophil count (cells/mL) × Monocyte count (cells/mL)/Lymphocyte count (cells/mL); SII = Neutrophil count (cells/mL) × Platelet count (cells/mL)/Lymphocyte count (cells/mL).

### 2.3 Definitions of covariates

Covariates primarily comprised demographic characteristics and established stroke risk factors. Demographic characteristics included sex, age, race/ethnicity, education level, and marital status. Age was stratified into three groups: 18–39, 40–59, and ≥60 years. Race/ethnicity was classified into Mexican American, Non-Hispanic Black, Non-Hispanic White, and Other. Education level was categorized as follows: Junior Middle School or Below (9th–11th Grade, including 12th grade with no diploma, and Less Than 9th Grade), Senior High School or General Educational Development (High School Graduate/General Educational Development or Equivalent), and College or Above (Some College or Associate Degree, and College Graduate or Above). Marital status was classified as Married/Partnered (Married, Living with Partner), Separated/Widowed (Widowed, Divorced, Separated), and Single (Never Married). Smoking status was defined as “Yes” if a participant had smoked at least 100 cigarettes in their lifetime; otherwise, it was classified as “No.” Alcohol consumption was defined as “Yes” if a participant had consumed at least 12 alcoholic drinks in any given year; otherwise, it was classified as “No.” Body mass index (BMI) (20), hypertension (21), diabetes (22), and coronary heart disease (23) all established stroke risk factors, were included as covariates. Hypertension was defined as a self-reported physician diagnosis or current use of antihypertensive medication. Diabetes and coronary heart disease were defined based on self-reported physician diagnoses.

### 2.4 Statistical analysis

First, we organized the data for analysis. Categorical variables were presented as counts and proportions, and the chi-square test was employed to compare intergroup differences. Quartile-based adjustments and analyses were conducted for the CALLY index. Subsequently, three weighted generalized linear regression models were applied to examine the association between the CALLY index and stroke: Model 1 (unadjusted), Model 2 (adjusted for sex, race, and age), and Model 3 (adjusted for sex, race, age, BMI, education level, marital status, smoking status, alcohol consumption, hypertension, diabetes, and coronary heart disease). Finally, restricted cubic splines (RCS) with three knots were employed to investigate the potential non-linear association between the CALLY index and stroke. Segmented regression models were utilized to evaluate the relationship and determine its linearity. Additionally, area under the curve (AUC) and receiver operating characteristic (ROC) curve analyses were performed to assess the predictive capability of the CALLY index and other inflammatory markers, including SIRI and SII. The accuracy and effectiveness of these markers in distinguishing different clinical or disease states were assessed using AUC and ROC curve analyses. All analyses were conducted using R (version 4.3.3).

## 3 Results

### 3.1 Baseline characteristics

**Figure 2:**
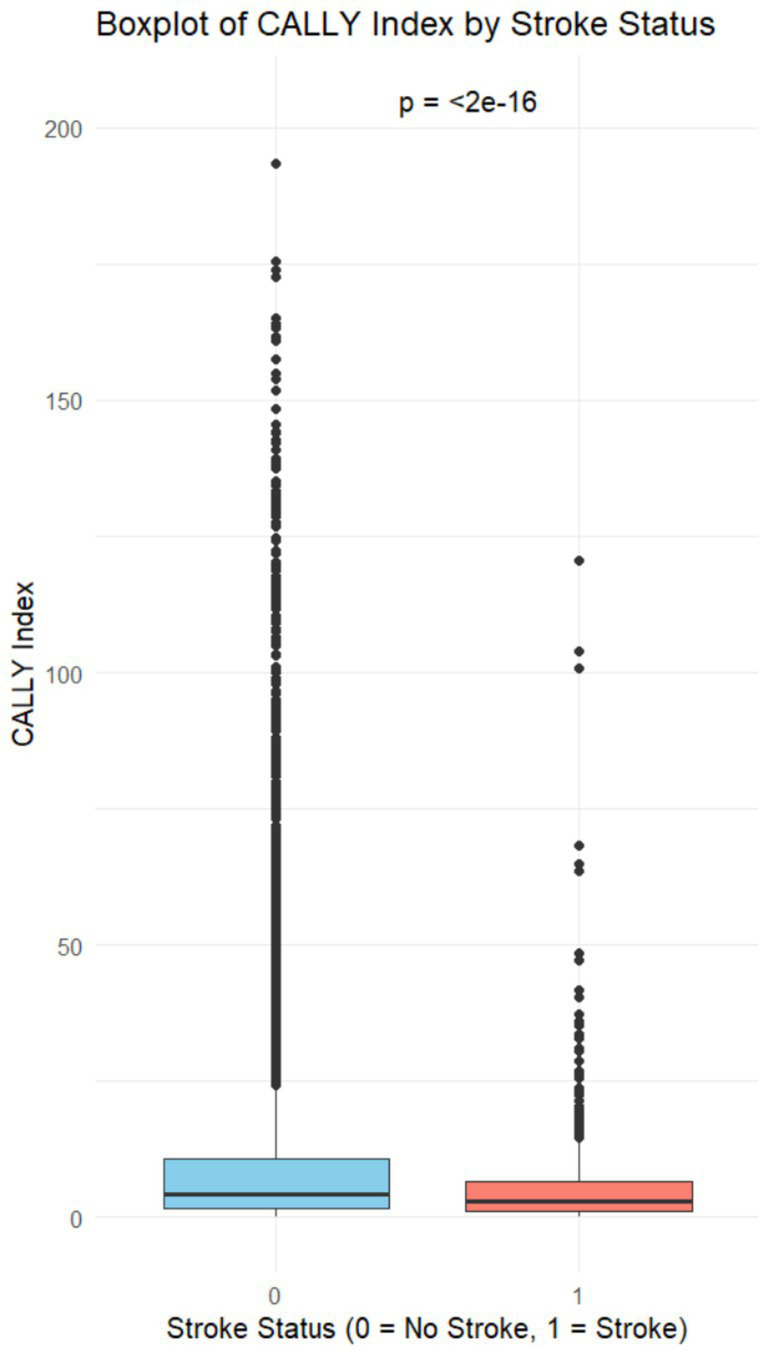
Boxplot of CALLY index by stroke status.

This study included 17,511 individuals from the NHANES database across 6 cycles (1999–2010). The mean age was 49.53 ± 18.3 years, with males comprising 49.0% and females 51.0%. The weighted demographic and laboratory characteristics of participants, stratified by stroke status (stroke: 637; no stroke: 16,876), revealed significant differences in age (66.96 ± 13.58 vs. 48.87 ± 18.13, *p* < 0.001) and BMI (29.64 ± 6.36 vs. 28.83 ± 6.62, *p* = 0.003) between stroke and non-stroke individuals. Significant differences were also observed in race, education level, marital status, smoking status, alcohol consumption, hypertension, and coronary heart disease (all *p* < 0.001). Detailed information is provided in Table 1. In the box plot, the maximum CALLY index in the non-stroke group was significantly higher than that in the stroke group (*p* < 0.001), suggesting a potential association between a higher CALLY index and a reduced risk of stroke (Figure 2).

**Table 1:** Baseline characteristics.

Characteristics	Total (*n* = 17,511)	Non-stroke (*n* = 16,874)	Stroke (*n* = 637)	*P*
Age Group, *n*(%)				<0.001^*^
18–39	6,041 (34.50)	6,013 (35.63)	28 (4.40)	
40–59	5,534 (31.60)	5,408 (32.05)	126 (19.78)	
≥60	5,936 (33.90)	5,453 (32.32)	483 (75.82)	
Race, *n*(%)				<0.001^*^
Mexican American	3,329 (19.01)	3,258 (19.31)	71 (11.15)	
Non-Hispanic black	3,302 (18.86)	3,160 (18.73)	142 (22.29)	
Non-Hispanic white	8,913 (50.90)	8,534 (50.57)	379 (59.50)	
Other	1,967 (11.23)	1,922 (11.39)	45 (7.06)	
Education, *n*(%)				<0.001^*^
Colleges or above	8,381 (47.86)	8,150 (48.30)	231 (36.26)	
Junior middle schools or below	4,924 (28.12)	4,676 (27.71)	248 (38.93)	
Senior high schools or GED	4,206 (24.02)	4,048 (23.99)	158 (24.80)	
Marital, *n*(%)				<0.001^*^
Married/partnered	10,822 (61.80)	10,466 (62.02)	356 (55.89)	
Separated/widowed	3,877 (22.14)	3,638 (21.56)	239 (37.52)	
Single	2,812 (16.06)	2,770 (16.42)	42 (6.59)	
BMI, *n*(%)				0.002^*^
<25	5,163 (29.48)	5,009 (29.68)	154 (24.18)	
≥30	6,261 (35.75)	5,996 (35.53)	265 (41.60)	
25–30	6,087 (34.76)	5,869 (34.78)	218 (34.22)	
Smoke, *n*(%)				<0.001^*^
No	9,140 (52.20)	8,895 (52.71)	245 (38.46)	
Yes	8,371 (47.80)	7,979 (47.29)	392 (61.54)	
Alcohol, *n*(%)				<0.001^*^
No	5,127 (29.28)	4,891 (28.99)	236 (37.05)	
Yes	12,384 (70.72)	11,983 (71.01)	401 (62.95)	
Hypertension, *n*(%)				<0.001^*^
No	11,569 (66.07)	11,421 (67.68)	148 (23.23)	
Yes	5,942 (33.93)	5,453 (32.32)	489 (76.77)	
Diabetes, *n*(%)				<0.001^*^
No	15,531 (88.69)	15,102 (89.50)	429 (67.35)	
Yes	1980 (11.31)	1772 (10.50)	208 (32.65)	
CHD, *n*(%)				<0.001^*^
No	16,757 (95.69)	16,231 (96.19)	526 (82.57)	
Yes	754 (4.31)	643 (3.81)	111 (17.43)	
CALLY quantile, *n*(%)				<0.001^*^
Q1	4,374 (24.98)	4,152 (24.61)	222 (34.85)	
Q2	4,381 (25.02)	4,212 (24.96)	169 (26.53)	
Q3	4,378 (25.00)	4,235 (25.10)	143 (22.45)	
Q4	4,378 (25.00)	4,275 (25.33)	103 (16.17)	

Data presented are mean ± SD or *n* (%). BMI, body mass index; CHD, coronary heart disease; CALLY, C-reactive protein-albumin-lymphocyte index. Asterisks indicated *p*-values of statistical significance. Bold values and asterisks indicate statistically significant *P*-values.

### 3.2 Relationship between CALLY index and stroke

**Figure 3:**
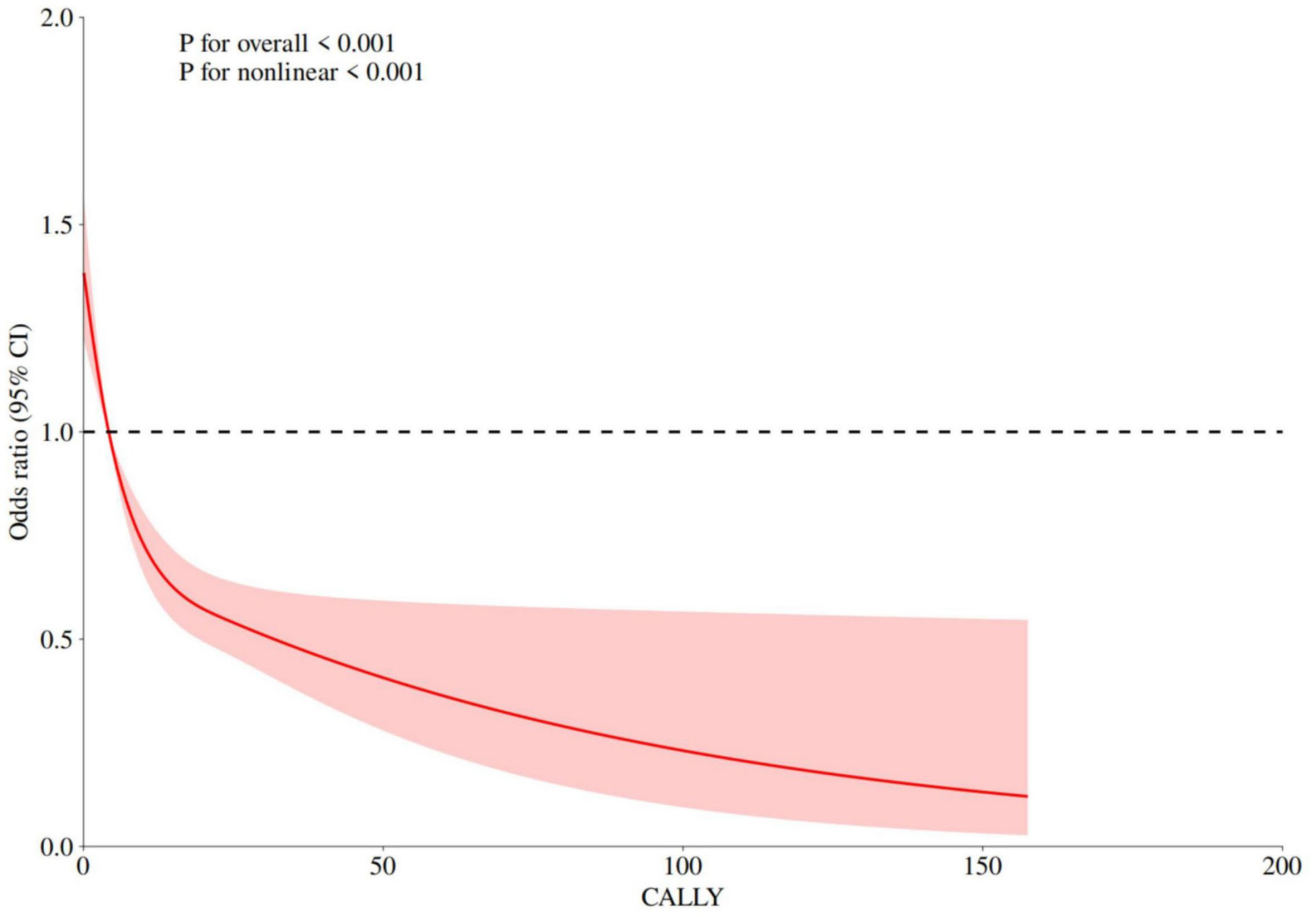
RCS analysis of relationship between the CALLY index and stroke.

Three logistic regression models were developed to investigate the independent association between the CALLY index and stroke. In Model 1 (unadjusted), a higher CALLY index was associated with a lower risk of stroke (OR: 0.97, 95% CI: 0.96–0.98, *p* < 0.001). In Model 2, after adjustment for sex, race, and age, the findings remained consistent with Model 1 (OR: 0.99, 95% CI: 0.98–0.99, *p* = 0.003). In Model 3, after further adjustment for BMI, education level, marital status, smoking status, alcohol consumption, hypertension, diabetes, and coronary heart disease, a higher CALLY index remained associated with a lower risk of stroke (OR: 0.99, 95% CI: 0.98–0.99, *p* = 0.045) (Table 2). Thus, each 1 SD increase in the CALLY index was associated with a 1% reduction in stroke risk. Additionally, the CALLY index was categorized into quartiles for analysis. In Model 3, participants in Q4 had a 25% lower likelihood of stroke than those in Q1 (OR: 0.75, 95% CI: 0.58–0.97, *p* = 0.030). Similarly, participants in Q3 had a 22% lower likelihood of stroke than those in Q1 (OR: 0.78, 95% CI: 0.62–0.98, *p* = 0.036) (Table 2). Finally, weighted RCS analysis was conducted to model and visualize the association between the CALLY index and stroke risk. The analysis revealed a non-linear, L-shaped association between the CALLY index and stroke risk (*p* < 0.001) (Figure 3).

**Table 2:** Weighted multivariate logistic analysis CALLY and stroke.

	Model 1		Model 2	Model 3		
	OR (95%CI)	*P*	OR (95%CI)	*P*	OR (95%CI)	*P*
CALLY	0.97 (0.96~0.98)	<0.001^*^	0.99 (0.98~0.99)	0.003^*^	0.99 (0.98~0.99)	0.045^*^
CALLY quantile						
Q1	1.00 (Reference)		1.00 (Reference)		1.00 (Reference)	
Q2	0.75 (0.61~0.92)	0.006^*^	0.74 (0.60~0.91)	0.005^*^	0.80 (0.65~0.99)	0.042^*^
Q3	0.63 (0.51~0.78)	<0.001^*^	0.68 (0.55~0.85)	<0.001^*^	0.78 (0.62~0.98)	0.036^*^
Q4	0.45 (0.36~0.57)	<0.001^*^	0.65 (0.51~0.82)	<0.001^*^	0.75 (0.58~0.97)	0.030^*^

The association between the CALLY index and stroke among all participants in the NHANES cohort. Model 1 was an unadjusted crude model; Model 2 was built upon Model 1 by incorporating adjustments for age, gender, race. Model 3 further extended Model 2 by incorporating adjustments for the BMI, education level, marital status, smoking, drinking, hypertension, diabetes, and coronary heart disease. Asterisks indicated *P*-values of statistical significance. CALLY, C-reactive protein-albumin-lymphocyte index.

### 3.3 Subgroup analysis

To assess the consistency of the association between the CALLY index and stroke across different subgroups, we performed subgroup analyses stratified by age, race, marital status, hypertension, diabetes, and coronary heart disease. The results revealed no significant interactions among these variables (*p*: 0.064–0.523) (Table 3), suggesting that the association between the CALLY index and stroke was not substantially modified by these covariates, thereby reinforcing the robustness of the findings.

**Table 3:** Subgroup analysis.

Variables	OR (95%CI)	*P*	*P* for interaction
Age Group			0.532
18–39	1.00 (0.98~1.02)	0.743	
40–59	0.98 (0.96~1.00)	0.056	
≥60	0.99 (0.97~1.00)	0.019^*^	
Sex			0.109
Female	0.98 (0.97~0.99)	<0.001^*^	
Male	0.96 (0.95~0.98)	<0.001^*^	
Race			0.081
Mexican American	0.99 (0.97~1.01)	0.543	
Non-Hispanic black	0.98 (0.96~0.99)	0.010^*^	
Non-Hispanic white	0.96 (0.95~0.98)	<0.001^*^	
Other	0.99 (0.96~1.01)	0.220	
Education			0.130
Colleges or above	0.98 (0.96~0.99)	<0.001^*^	
Junior middle schools or below	0.98 (0.97~0.99)	0.008^*^	
Senior high schools or GED	0.95 (0.93~0.98)	<0.001^*^	
Marital			0.069
Married/partnered	0.97 (0.95~0.98)	<0.001^*^	
Separated/widowed	0.99 (0.97~1.00)	0.060	
Single	0.99 (0.97~1.01)	0.183	
BMI			0.998
<25	0.97 (0.96~0.99)	<0.001^*^	
≥30	0.97 (0.95~1.00)	0.043^*^	
25–30	0.97 (0.96~0.99)	0.003^*^	
Smoke			0.417
No	0.98 (0.96~0.99)	<0.001^*^	
Yes	0.97 (0.96~0.98)	<0.001^*^	
Alcohol			0.923
No	0.97 (0.96~0.99)	0.001^*^	
Yes	0.97 (0.96~0.99)	<0.001^*^	
Hypertension			0.163
No	0.98 (0.96~0.99)	0.005^*^	
Yes	0.99 (0.98~1.00)	0.070	
Diabetes			0.064
No	0.97 (0.96~0.98)	<0.001^*^	
Yes	0.99 (0.98~1.01)	0.283	
CHD			0.099
No	0.97 (0.96~0.98)	<0.001^*^	
Yes	0.99 (0.97~1.02)	0.604	

Subgroup analyses stratified by age, sex, race, education, martial, BMI, smoke, alcohol, hypertension, diabetes and CHD. BMI, body mass index; CHD, coronary heart disease. Asterisks indicated *P*-values of statistical significance.

### 3.4 ROC analysis

**Figure 4:**
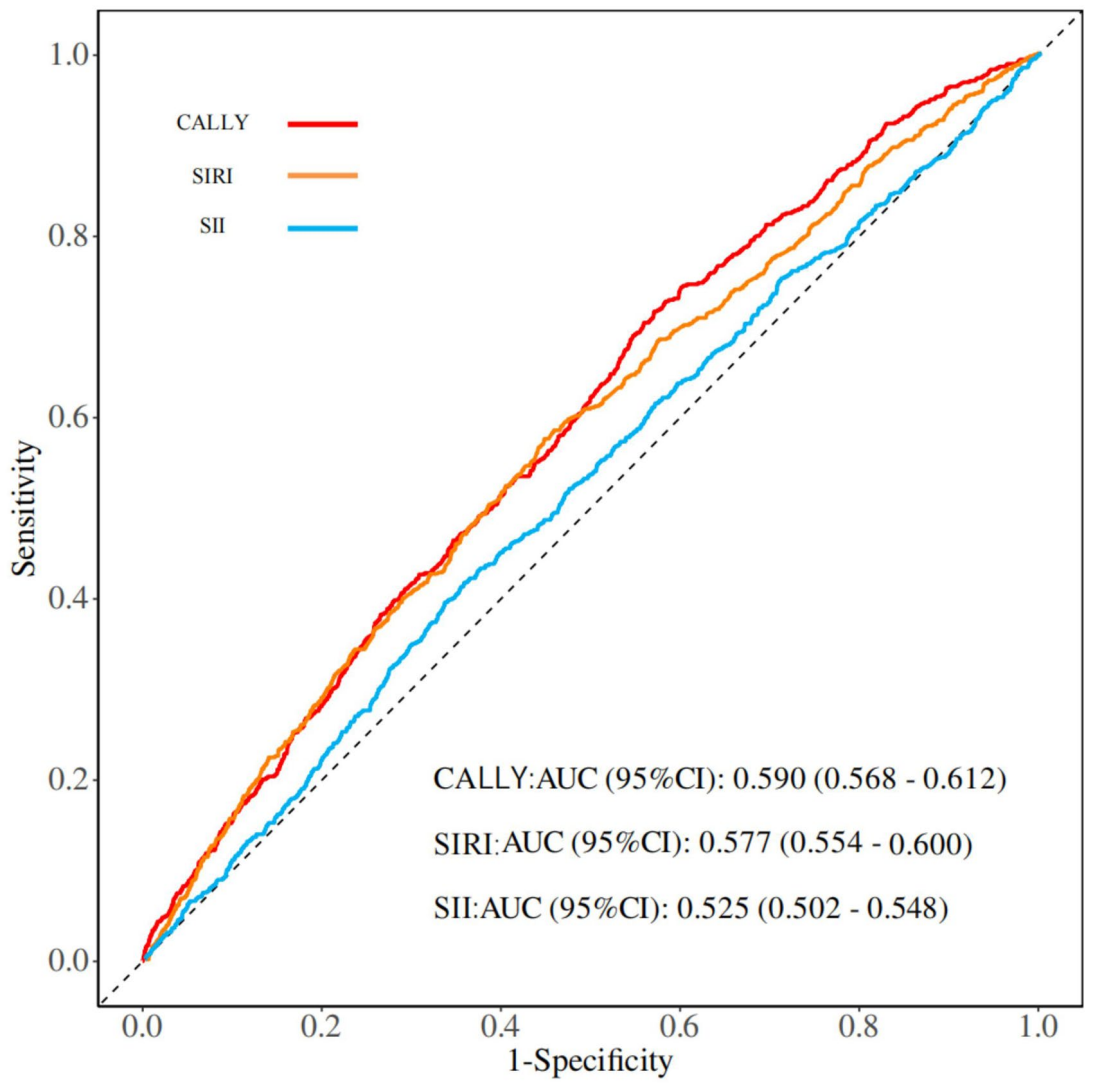
ROC analysis of the CALLY index, SIRl index, and Sll index.

**Figure 5:**
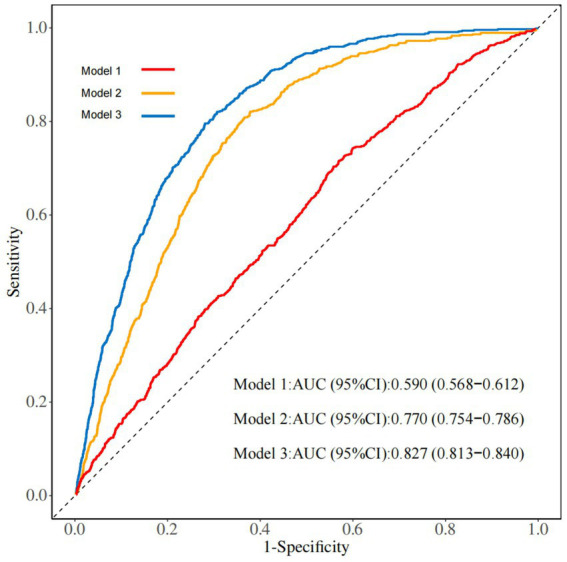
ROC analysis of the three models of the CALLY index.

Sensitivity analyses were performed to assess the predictive ability of the CALLY, SIRI, and SII indices for stroke. The ROC curve analysis showed that the AUC value of the CALLY index (0.590, 95% CI: 0.568–0.612) was higher than that of the SIRI (0.577, 95% CI: 0.554–0.600) and SII indices (0.525, 95% CI: 0.502–0.548) (Figure 4). These findings suggest that the CALLY index exhibits greater stability and accuracy in predicting stroke risk compared to the SIRI and SII indices. Additionally, ROC analysis of the CALLY index across three models revealed that the AUC for Model 2 was 0.770 (95% CI: 0.754–0.786), whereas for Model 3, it was 0.827 (95% CI: 0.813–0.840) (Figure 5).

## 4 Discussion

This study aimed to assess the association between the CALLY index and stroke risk in U.S. adults (≥18 years) from 1999 to 2010. We found that the CALLY index was significantly elevated in individuals with stroke compared to those without. Furthermore, RCS analysis identified a potential nonlinear L-shaped inverse association between the CALLY index and stroke risk. This phenomenon may be attributed to the fact that elevated levels of albumin and lymphocytes could suppress inflammatory damage through mechanisms such as antioxidant activity and immune modulation. However, once these levels surpass a certain threshold, their protective effects no longer exhibit linear enhancement, leading to a plateau in risk within higher index ranges. Finally, subgroup and ROC analyses further confirmed the robustness and predictive accuracy of the results. Additionally, comparisons with other inflammatory markers suggested that the CALLY index demonstrated superior stability and predictive accuracy for stroke risk.

During the pathological processes of both IS and HS, the body undergoes a series of immune-inflammatory responses. In ischemic stroke, early ischemia and hypoxia result in brain tissue damage, energy metabolism disorders, and blood–brain barrier disruption, leading to neuronal death and the formation of an ischemic penumbra (24, 25).In hemorrhagic stroke, vascular rupture causes blood components to extravasate directly into the brain, forming hematomas that result in structural damage (26). Following stroke onset, cells affected by the initial injury trigger a rapid cascade of events, leading to an intense inflammatory response.

A cross-sectional study based on the 2005–2018 NHANES database, including 902 stroke patients, suggested that an elevated SII index may be associated with a higher risk of ischemic stroke (27). Additionally, a cross-sectional study of stroke patients within a hypertensive population, based on the 1999–2020 NHANES database, demonstrated that higher SIRI index levels were significantly associated with an increased incidence of stroke in hypertensive patients (28). Both studies suggest that lymphopenia during stroke may reflect immune dysfunction. This phenomenon may be explained by the distinct roles of T and B lymphocytes in stroke pathophysiology. Studies indicate that following IS onset, CD8+ cytotoxic T lymphocytes infiltrate the ischemic site within 3 h. These cells induce direct neuronal damage by releasing cytotoxic proteins, including granzyme and perforin. Furthermore, they secrete inflammatory mediators, including IL-16, which promote the recruitment of immune cells and exacerbate damage to the vascular endothelium and blood–brain barrier (29, 30). In contrast, B cells can inhibit the activation and recruitment of T cells, macrophages, and other immune cells, thereby reducing the extent of cerebral infarction and improving neurological deficits (31). In HS, lymphocytes are also critical mediators. Evidence suggests that lymphocytes regulate local inflammatory responses by secreting cytokines, modulating blood–brain barrier permeability, and ameliorating neurological symptoms following brain hemorrhage (32).

A prospective cohort study investigating the effects of C-reactive protein (CRP) levels and dyslipidemia on stroke reported that elevated CRP levels and dyslipidemia were associated with an increased stroke risk in men (33).Similarly, studies have indicated that serum CRP levels rise significantly 48–72 h post-admission in patients with HS, with the magnitude of elevation correlating with hematoma volume (34).As an inflammatory marker, CRP levels rise rapidly during stroke, likely in response to immune activation triggered by ischemic or hemorrhagic brain injury. This cascade activates inflammatory cells and promotes cytokine release, which in turn stimulates hepatic CRP synthesis (35, 36).

A strong association has been observed between albumin levels and IS. A study conducted in a cohort of 2,986 individuals from North Manhattan reported that low serum albumin levels were associated with an increased risk of IS, particularly in cases of cardiogenic embolism and cryptogenic subtypes (37). Similarly, a prospective study examining the association between serum albumin levels and clinical outcomes in IS patients reported that each 1 g/L decrease in serum albumin was linked to a 3% increase in the risk of poor functional prognosis and a 7% increase in mortality risk (38). In patients with HS, studies have suggested that hypoalbuminemia may be associated with an elevated risk of HS and a higher incidence of adverse events in its prognosis. These findings indicate that albumin, as a nutritional marker, reflects both an individual’s overall health status and systemic inflammation levels (39). The potential mechanisms may involve albumin’s antioxidant, anti-inflammatory, and endothelial-protective properties (40–42). Compared with the SIRI and SII indices, the CALLY index exhibits greater stability and accuracy in predicting stroke risk, potentially due to its dependence on serum albumin levels.

Our study has several strengths. First, we not only analyzed the predictive performance of the CALLY index for stroke but also compared its predictive ability with that of the SII and SIRI indices. Second, we adjusted for covariates in this study, constructed multiple predictive models, and performed sensitivity analyses to ensure the robustness of the final results. However, this study has several limitations. First, as a cross-sectional study, this research identifies an association between the CALLY index and stroke, but the causal relationship remains unclear. Second, as the NHANES database collects data from a representative U.S. population, the findings may not be generalizable to the global population. Furthermore, the reliance on self-reported diagnoses for case inclusion could introduce selection bias, while unmeasured covariates (e.g., genetic susceptibility, socioeconomic status) might act as residual confounders, potentially influencing the observed associations. Finally, although the CALLY index demonstrates better predictive performance than other inflammatory markers, its overall clinical utility remains limited, necessitating further investigation in future studies.

## 5 Conclusion

A reduced CALLY index may be linked to a higher risk of stroke. Furthermore, the CALLY index demonstrates superior predictive performance compared to the SIRI and SII indices. The association between the CALLY index and stroke risk provides valuable insights for future stroke prevention and management strategies.

## Data Availability

Publicly available datasets were analyzed in this study. This data can be found at: https://www.cdc.gov/nchs/nhanes.

## Footnotes

^1^ https://www.cdc.gov/nchs/nhanes
